# Effectiveness and Cost-Effectiveness of Antidepressants in Primary Care: A Multiple Treatment Comparison Meta-Analysis and Cost-Effectiveness Model

**DOI:** 10.1371/journal.pone.0042003

**Published:** 2012-08-02

**Authors:** Joakim Ramsberg, Christian Asseburg, Martin Henriksson

**Affiliations:** 1 Department of Learning, Informatics, Management and Ethics, Medical Management Center, Karolinska Institutet, Stockholm, Sweden; 2 ESiOR Oy, Kuopio, Finland; 3 AstraZeneca Nordic, Södertälje, Sweden; National Institute for Public Health and the Environment, The Netherlands

## Abstract

**Objective:**

To determine effectiveness and cost-effectiveness over a one-year time horizon of pharmacological first line treatment in primary care for patients with moderate to severe depression.

**Design:**

A multiple treatment comparison meta-analysis was employed to determine the relative efficacy in terms of remission of 10 antidepressants (citalopram, duloxetine escitalopram, fluoxetine, fluvoxamine mirtazapine, paroxetine, reboxetine, sertraline and venlafaxine). The estimated remission rates were then applied in a decision-analytic model in order to estimate costs and quality of life with different treatments at one year.

**Data Sources:**

Meta-analyses of remission rates from randomised controlled trials, and cost and quality-of-life data from published sources.

**Results:**

The most favourable pharmacological treatment in terms of remission was escitalopram with an 8- to 12-week probability of remission of 0.47. Despite a high acquisition cost, this clinical effectiveness translated into escitalopram being both more effective and having a lower total cost than all other comparators from a societal perspective. From a healthcare perspective, the cost per QALY of escitalopram was €3732 compared with venlafaxine.

**Conclusion:**

Of the investigated antidepressants, escitalopram has the highest probability of remission and is the most effective and cost-effective pharmacological treatment in a primary care setting, when evaluated over a one year time-horizon. Small differences in remission rates may be important when assessing costs and cost-effectiveness of antidepressants.

## Introduction

Current guidelines for the pharmacological treatment of moderate to severe major depressive disorder uniformly recommend generic formulations of selective serotonin reuptake inhibitors (SSRI) as first line treatment [1]–[4].

However, systematic reviews have found evidence that some of the newer additions to the therapeutic arsenal– in particular the SSRI escitalopram and the serotonin nor-adrenaline reuptake inhibitor (SNRI) venlafaxine– may have a somewhat better efficacy than other second-generation antidepressants [2], [5], [6]. As the newer treatment options are generally more expensive than drugs for which generic formulations are available, potential improvement in efficacy has to be balanced against treatment costs. This is particularly important as untreated or poorly treated depression is costly for societies around the world [7], suggesting that relatively small differences in efficacy may offset a higher acquisition cost.

An important contribution by Cipriani et al [8] began to address this issue and found mirtazapine, escitalopram, venlafaxine and sertraline to be the most efficacious treatments when pooling the results from a large number of trials in a multiple treatment comparison meta-analysis. When jointly considering response and also acceptability, Cipriani et al found escitalopram and sertraline to have the best profile of both efficacy and acceptability leading to the tentative conclusion that the generically available sertraline is likely to be cost-effective given its lower acquisition cost.

Although this and other studies have gone to some length determining treatment outcome on a disease specific endpoint, such as response, studies investigating how these clinical findings impact both costs and health outcomes are at present lacking.

Therefore, we undertook a systematic review to estimate the efficacy in terms of remission rates of different antidepressants in a primary care setting utilizing randomized evidence. Furthermore, we sought to determine the costs and a health outcome incorporating both quantity and quality of life (quality adjusted life years, or QALYs) associated with the estimated remission rates.

A Bayesian multiple treatment comparison, or network meta-analysis, was used to combine direct within-trial drug comparisons with indirect evidence from other trials as this offers the possibility of a more comprehensive synthesis of evidence. In addition, we wanted to use remission rather than the more frequently used response as our efficacy endpoint. There is substantial support in the literature that remission is the preferred endpoint due to its clinical relevance and close relation to resource utilization [9], [10]. We included tricyclic antidepressants (TCAs) in our analysis, not primarily because they are commonly used alternatives for first line treatment of depression but for broadening the evidence base of efficacy, as trials randomizing patients to TCAs with SSRIs or SNRIs as comparators provide important additional information on the efficacy of SSRI and SNRI.

In recent years, psychological treatment – especially cognitive behavioral therapy- have been put forward as first line treatment of depression in primary care (e.g. [2], [11]). Both psychological and pharmacologic therapies appear effective in the treatment of depressive disorders, and it is very important to be able to individualize treatment of depression given the idiosyncratic response and tolerability issues as well as the lack of patient adherence. The present study is not addressing the larger issue of an optimal long-term treatment algorithm for depression, but rather which pharmacological treatment is optimal to use as first line treatment in primary care for patients with moderate to severe depression.

## Methods

### Patients, Interventions and Outcomes

Costs and health outcomes of ten antidepressants considered relevant for first line treatment of patients with a diagnosis of major depressive disorder in primary care were compared in our decision analytic model: citalopram, duloxetine, escitalopram, fluoxetine, fluvoxamine mirtazapine, paroxetine, reboxetine, sertraline and venlafaxine. The main outcomes were costs and quality-adjusted survival at one year follow-up where costs were evaluated from a societal perspective. The meta-analysis included seven additional antidepressants, which are currently not considered to be alternatives for first line treatment of MDD in primary care:amitriptyline, dosulepin, imipramine, lofepramine, maprotiline, milnacipran, and nortriptyline.

Due to the lack of long-term data and complexities of modelling disease progression and treatment sequences over a long period a relatively short time horizon was chosen for the cost-effectiveness analysis. While this is inevitably a simplification, a one-year model will capture the majority of costs and health outcomes associated with the acute treatment of depression.

**Figure 1:**
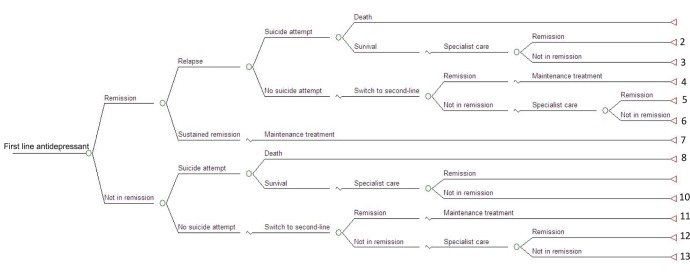
Decision tree structure.

Meta-analytic techniques were used to synthesize data on remission rates at 8–12 weeks from randomized controlled trials (RCT). Costs and QALYs for each treatment strategy were then determined by combining the estimated remission rates with data on relapse, risk of suicide, costs and health-related quality of life in a decision-analytic model. The structure of the decisions-tree model is shown in Figure 1.

### Data Sources

Table S1 gives a detailed description of data sources for the model parameters.

### Evidence Synthesis

In the systematic review undertaken to establish remission rates, we included head-to-head RCTs comparing at least two antidepressants as mono-therapy in the treatment of adults with unipolar major depression. Study medications could come from any of the groups TCA, SSRI, monoamine oxidase (MAO)-inhibitors, Alpha-2-antagonists, SNRI or nor-adrenaline re-uptake inhibitors (NRI). Minimum study duration was 6 weeks. Although 8–12 weeks treatment is more clinically relevant and was used in a sensitivity analysis, 6 week studies are very common and also informative of relative efficacy. Remission had to be reported as primary or secondary endpoint defined as a Hamilton Depression Rating Scale (HAMD)-score < = 7 (for a few studies a score of 8 was used) or as a Montgomery-Åsberg Depression Rating Scale (MADRS)-score < = 12. Studies enrolling adult patients with a diagnosis of moderate to severe major depression (a MADRS-score of 18 or more or a HAMD-score of 15 or more) were included in the analysis. Studies not excluding patients with psychiatric co-morbidities such as bipolar disorder, substance abuse or psychosis were deemed unsuitable.

We searched the electronic databases PubMed, PsychLit and the Cochrane Central Register of Controlled Trials (last search on January 25, 2010). Search terms included combinations of “Antidepressive Agents” [MeSH], names of specific antidepressive agents and “Depressive Disorder” [MeSH]. Searches were limited to randomized controlled trials, humans and all adults. Also, searches were limited to citations entered in PubMed after 2005 since earlier systematic reviews had searched the literature prior to that date for all antidepressive agents of interest except mirtazapine and reboxetine [12]. Searches for the latter two were not limited in time. Reference lists from previously published systematic reviews were hand searched. We asked pharmaceutical companies to submit dossiers with both published and unpublished studies. Furthermore, we searched the depositories for clinical trials www.clinicalstudyresults.org and www.centerwatch.com/patient/trials in addition to the manufacturers’ own web pages for unpublished studies.

We used a pre-specified data abstraction form and from the studies we extracted data on intervention, control, number of patients, dosage, study duration, study setting (in- or out-patient), location, sponsor and patients’ sex, age, weight, HAMD and MADRS scores at baseline. The efficacy and tolerability data that were extracted amounted to definition of remission used, number of patients in remission, number of completers, number of dropouts due to adverse events, and lack of efficacy. One reviewer extracted data and each data point was independently checked by another reviewer. Any disparities were resolved by consensus.

Classical methods for meta-analysis focus on comparing two treatments, e.g. drug A against drug B, or drug A against placebo [13]. However, these methods are somewhat limiting when more than two treatments are to be compared or when certain treatments have not been studied head-to-head [14]. Network treatment comparison (also known as mixed treatment comparison, MTC), a Bayesian meta-analysis method, offers the possibility of a more comprehensive synthesis of evidence [14]. Therefore a Bayesian MTC was used to combine direct within-trial drug comparisons with indirect evidence from other trials. The indirect comparisons, which preserve the within-trial randomization, were constructed from trials that had at least one treatment in common.

In particular, the outcome, i.e. probability of remission, was modelled on the log-odds scale with additive fixed effects for each treatment. A random-baseline model was employed to account for heterogeneity between trials. Writing m_d_ for the log-odds effect of treatment d relative to the reference treatment, and denoting by p_it_ the probability of remission in arm i of trial t, where r_it_ of n_it_ patients experienced remission under treatment d_it_ in that trial arm and mu_t_ denotes the trial baseline, this model is defined by equations:


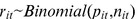
(1)


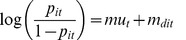
(2)

Uninformative priors were used on all unknown quantities. The model was evaluated using WinBUGS 1.4.3 [15].

Heterogeneity was assessed by calculating Q and I^2^ statistics of heterogeneity using a standard pairwise meta-analysis on the log odds ratios. To assess the inconsistency in the network meta-analysis, the node-splitting techniques explained in Dias et al [16] have been used. That is, for each pairwise comparison between X and Y where both direct and indirect evidence exist, an alternative meta-analytic model is evaluated that summarises the difference between these two pieces of evidence in terms of an additional quantity, omega_XY. Dias et al define a “P-value” which can help identify those pairwise comparisons where inconsistency is high. Additionally, the Bayesian Deviance Information Criterion (DIC) can be used to find out whether a model that splits a given pairwise comparison into the direct and indirect evidence would provide better model fit than the model without such splits.

### Relapse and Risk of Suicide

Data on probability of relapse after remission were obtained from a systematic review of observational studies in primary care [17]. The average time until remission and relapse as well as the effect of second and third line treatment were taken from a large naturalistic outcomes study of sequenced treatment of depression in primary care [18]–[20]. According to this study venlafaxine was the most efficacious second-line treatment, and we assumed that all patients who fail in the first line of treatment were treated with venlafaxine.

We obtained the suicide risk from a study of FDA data [21].

### Costs

All costs are expressed in Euros (€) at 2009 prices. Because of the one-year time horizon, no discounting was used.

Medication costs were obtained from the price data base at the Dental and Pharmaceutical Benefits Agency (TLV) (www.tlv.se) using appropriate dosing of the drugs from a panel of clinicians. These costs are shown in Table 1.

**Table 1:** Pharmaceutical costs and other model parameters.

*Pharmaceutical costs per month (assumed daily dose)*		*Parameter distribution*
Mirtazapine (30 mg)	1.60 €	Deterministic
Escitalopram (10 mg)	21.80 €	Deterministic
Sertraline (50 mg)	0.60 €	Deterministic
Paroxetine (20 mg)	2.20 €	Deterministic
Duloxetine (60 mg)	36.70 €	Deterministic
Reboxetine (8 mg)	30.30 €	Deterministic
Venlafaxine (150 mg)	3.10 €	Deterministic
Citalopram (20 mg)	1.70 €	Deterministic
Fluoxetine (20 mg)	2.20 €	Deterministic
Fluvoxamine (100 mg)	23.10 €	Deterministic
Second line treatment	3.10 €	Deterministic
(Venlafaxine)		
***Direct cost in depression/remission (per month)***		
Depression in primary care	374 €	Gamma(91.96, 0.2459)
Remission in primary care	273 €	Gamma(105.29, 0.4461)
Depression in specialist care	784 €	Normal(2, 0.25)*
Remission in specialist care	546 €	Normal(2, 0.25)*
***Societal cost (direct + indirect) in depression/remission (per month)***		
Depression in primary care	1 185 €	Gamma(208.84, 0.1762)
Remission in primary care	715 €	Gamma(151.97, 0.2125)
Depression in specialist care	2 370 €	Normal(2, 0.25)*
Remission in specialist care	1 430 €	Normal(2, 0.25)*
Loss of production deceased	3 548 €	Deterministic
***Other costs (one-off costs)***		
Dying of suicide attempt	3 495 €	Gamma(12.22, 0.0035)
Treatment switch	150 €	Deterministic
Suicide attempt	11 753€	Gamma(5.52, 0.0005)
***Health-related quality of life***		
QALY-weight remission	0.81	Beta(312, 72)
Decrement QALY-weight in depression	0.24	Gamma(144, 600)
***Transition probabilities***		
Remission after switch	0.248	Beta(24.8, 75.2)
Relapse	0.110	Beta(11, 89)
Suicide attempt in depression	0.031	Beta(3.1, 96.9)
Die of suicide attempt	0.110	Beta(11, 89)
Remission after specialist care	0.248	Beta(24.8, 75.2)

All costs are in Euro (€) in 2009 prices. Exchange rate used to convert Swedish prices to Euros was €1 =  SEK 10.

* Uncertainty in this estimate is incorporated as a normal distribution around the estimated increase in costs in specialist care compared with primary care.

Costs of primary care patients being in the states remission and no remission were available from a prospective observational study following 447 patients for six months in a Swedish primary care setting [10]. Total costs, including productivity losses, were estimated for patients in remission and patients not in remission thus providing appropriate estimates for the decision-analytic model. These data were corroborated by another Swedish primary care study which had used response rather than remission as endpoint [22]. Costs for specialist care were obtained from a previous study which utilized a Swedish expert panel to quantify resource use [23] and costs associated with suicide. We assumed that the ratio of costs for patients in remission and not in remission in specialist care was the same as in primary care, i.e. 39% lower costs for patients in remission. Costs associated with suicide attempts were available from a Swedish study of 97 suicide attempts [24]. The cost estimates are summarized in Table 1.

### Quality of Life

Quality-of-life weights, by remission status, measured using EQ-5D were obtained from the same Swedish observational study investigating costs [10] and are shown in Table 1.

### Costs-effectiveness

The analysis was undertaken from a societal perspective. Mean costs and QALYs for all treatment strategies are presented and their cost-effectiveness compared using standard decision rules and estimating incremental cost-effectiveness ratios (ICERs) as appropriate. The ICER should be interpreted as the additional cost required to achieve an additional unit of health outcome (QALY) when providing one treatment over another.

### Alternative Scenarios

In the base-case meta-analysis all out-patient studies with eight to twelve weeks duration were included because this corresponds most closely to clinical practice. Alternative selections of studies were also investigated. One analysis included all studies with six weeks or longer duration. Another scenario considered only studies with flexible dosing as antidepressants are used with flexible doses in clinical practice. A further analysis included in-patient studies. Finally, one analysis excluded studies using what may be considered inappropriate dosing (i.e. too high) of duloxetine.

Alternative scenarios were investigated also for the cost-effectiveness analysis. In one analysis the base case societal costs were replaced with direct health care costs as this is the relevant perspective for many decision makers. The risk of suicide is sometimes claimed to be very high, perhaps 15% [25], but systematic reviews have shown that for patients in primary care the risk may be 2–6% or lower [26], [27]. In an additional sensitivity analysis the suicide rates were varied between 0.02 and 0.15. Finally, the rate of relapse was varied as evidence is scarce for this parameter.

### Probabilistic Sensitivity Analysis

Probabilistic sensitivity analysis (PSA) using Monte Carlo simulations was used when analyzing the cost-effectiveness model [28]. In the PSA the uncertainty around single input parameter values are characterized by probability distributions. Gamma –distributions were used for costs, Beta-distributions for probabilities and QALY-weights, and gamma-distributions for decrements in QALY-weights (Table 1). The uncertainty around remission rates was derived from the results of the meta-analysis. In PSA, the uncertainty in all model inputs is evaluated simultaneously using simulation techniques. In the simulation parameter values are drawn randomly from all uncertain parameters and a cohort of hypothetical individuals is then run through the model and mean costs and health outcomes are calculated for all strategies. This procedure was repeated 5000 times, generating 5000 estimates of mean costs and mean effects of a cohort for both strategies. The results of the probabilistic analysis were also summarized as the probability of each treatment strategy being cost-effective at different willingness-to-pay values, or thresholds for cost-effectiveness, using cost-effectiveness acceptability curves.

The model was programmed and analysed in R version 2.13.1 (R Development Core Team (2011). R: A language and environment for statistical computing. R Foundation for Statistical Computing, Vienna, Austria. ISBN 3-900051-07-0, URL http://www.R-project.org/.) and WinBUGS 1.4.3 [15].

## Results

### Efficacy of Antidepressants

#### Study selection

**Figure 2:**
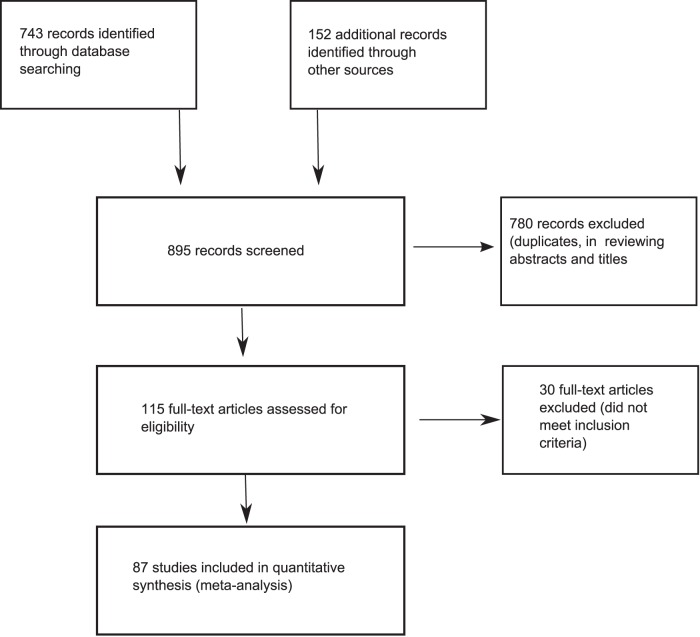
Flow diagram of literature search.

Nearly 900 citations were reviewed. After excluding studies that did not meet our inclusion criteria and duplicates, our data set included 87 studies with close to 20 000 patients (Figure 2). The search of PubMed, PsychLit and Cochrane Central Register of Controlled Trials databases provided a total of 743 citations. An additional 152 were identified when we hand searched reference lists from previously published systematic reviews, asked pharmaceutical companies to submit references and searched depositories for clinical trials for unpublished studies. Of these, 780 studies were discarded after reviewing the abstracts when it was clear that these papers did not meet the inclusion criteria. The full text of the remaining 115 citations was examined in detail. Thirty studies did not meet the inclusion criteria as described (they did not report remission or included patients with co-morbidities that were not allowed). Eighty-seven studies met the inclusion criteria and were included in the meta-analysis.

**Figure 3:**
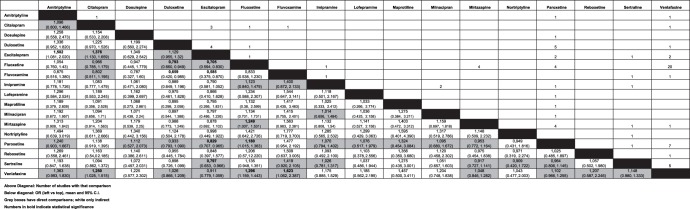
Evidence structure and estimated odds ratios. Figures above the diagonal show the number of studies with corresponding comparison. Figures below the diagonal show the odds ratio (left vs top), mean and 95% credibility intervals. Grey boxes indicate if direct comparisons were available; white boxes indicate odds ratios are based only on indirect comparison. Numbers in bold indicate at least 95% probability that estimate is different from 1.

The structure of the evidence is presented in Figure 3 and Table S2 gives an overview of the included studies.

Out of the 87 studies, 34 were unpublished or only available from conference proceedings or posters. A large majority of the studies were sponsored by a manufacturer of one or more of the study drugs (Table S2).

#### Probabilities of remission

The pair-wise odds ratios from the multiple treatment comparison are presented in Figure 3. In Table 2, the pair-wise odds ratios from the multiple treatment comparison are compared with odds ratios using traditional meta-analysis methods for those comparisons where two or more studies were available. It can be seen in Table 2 that the point estimates using different methods of pooling the studies are similar, but the level of precision is greatly increased when the full body of evidence is employed in the multiple treatment comparison. We note that the odds ratio for escitalopram against citalopram is lower in the MTC compared with pooling the head to head studies. The SSRI escitalopram is statistically significantly more likely to provide remission than amitriptyline, citalopram, fluoxetine, fluvoxamine, paroxetine, and sertraline.

**Table 2:** Odds ratios on remission using standard DerSimonian and Laird random-effects pair-wise meta-analysis and Bayesian MTC.

	Standard pair-wise meta-analysis		Bayesian multiple treatment comparison	
	Odds ratio	95 per cent CI	Odds ratio	95 per cent CrI
Venlafaxine vs. Fluoxetine	1.24	(1.09, 1.41)	1.29	(1.16, 1.44)
Paroxetine vs. Fluoxetine	1.14	(0.78, 1.67)	1.18	(1.02, 1.36)
Mirtazapine vs. Fluoxetine	1.07	(0.75,1.51)	1.25	(1.01, 1.51)
Paroxetine vs. Venlafaxine	0.83	(0.65, 1.05)	0.91	(0.80, 1.04)
Sertraline vs. Venlafaxine	0.83	(0.63, 1.09)	0.87	(0.75, 1.02)
Duloxetine vs. Paroxetine	1.07	(0.83, 1.38)	1.07	(0.92, 1.26)
Duloxetine vs. Escitalopram	0.95	(0.75, 1.20)	0.88	(0.76, 1.05)
Mirtazapine vs. Paroxetine	1.35	(0.98, 1.87)	1.05	(0.86, 1.30)
Citalopram vs. Escitalopram	0.66	(0.51, 0.85)	0.73	(0.60, 0.88)
Escitalopram vs.Venlafaxine	1.10	(0.75, 1.61)	1.10	(0.94, 1.28)
Escitalopram vs. Paroxetine	1.15	(0.59, 2.26)	1.21	(1.03, 1.41)

CI  =  confidence interval.

CrI  =  credibility interval.

#### Heterogeneity and consistency

When assessing inconsistency for the current network of evidence using node-splitting techniques [16], the only pairwise comparison with a P-value significant at the 0.05 level is that of fluvoxamine versus sertraline (p = 0.005), indicating inconsistency between the direct and indirect evidence in this particular comparison. According to the Bayesian Deviance Information Criterion analysis, in addition to fluvoxamine-sertraline, the splitting of the nodes citalopram-fluoxetine and amitriptyline-paroxetine could potentially also improve model fit. Despite evidence of some inconsistency it seems to be of limited magnitude and is improbable to alter the results significantly.

For the twelve pairs of treatments that were directly compared in more than one clinical study (i.e., involving in total 62 studies), we calculated the Q and I^2^ statistics of heterogeneity using a standard pairwise meta-analysis on the log odds ratios. Based on this, there was evidence of heterogeneity in one of the 12 comparisons (escitalopram versus paroxetine, two studies). One of these two studies may thus be considered an outlier, but it is unclear which one. From the inconsistency analysis, no inconsistency between direct and indirect evidence is found in this particular pairwise comparison. It is unlikely that one outlier in an evidence base on 87 studies will distort the results, and no heterogeneity correction was deemed necessary.

The derived probabilities of remission are shown in Table 3 for different selections of studies. The probabilities based on the meta-analysis using outpatient studies with 8–12 weeks duration are the key input into the decision-analytic model. The SSRI escitalopram has the highest probability of remission in all scenarios with a point estimate of 0.46 when all studies are included. Fluvoxamine has the lowest point estimate at 0.33.

**Table 3:** Estimated probability of remission in different scenarios.

	All studies	8–12 week	Outpatient 8–12	Studies with
		studies	week studies	flexible dose
Amitriptyline	0.362 (0.293–0.436)	0.421 (0.331–0.514)	0.435 (0.342–0.533)	n.a.
Citalopram	0.380 (0.332–0.430)	0.404 (0.352–0.456)	0.424 (0.364–0.485)	0.439 (0.364–0.517)
Dosulepine	0.401 (0.249–0.569)	n.a.	n.a.	0.434 (0.274–0.603)
Duloxetine	0.427 (0.384–0.471)	0.445 (0.397–0.493)	0.452 (0.401–0.504)	0.468 (0.413–0.526)
Escitalopram	0.456 (0.416–0.497)	0.471 (0.429–0.513)	0.487 (0.439–0.535)	0.509 (0.450–0.569)
Fluoxetine	0.371 (0.338–0.405)	0.390 (0.352–0.431)	0.400 (0.355–0.447)	0.404 (0.343–0.468)
Fluvoxamine	0.326 (0.242–0.420)	n.a.	n.a.	0.481 (0.319–0.640)
Imipramine	0.396 (0.332–0.464)	0.387 (0.312–0.465)	0.399 (0.305–0.500)	n.a.
Lofepramine	0.408 (0.257–0.575)	n.a.	n.a.	n.a.
Maprotiline	0.378 (0.183–0.604)	n.a.	n.a.	n.a.
Milnacipran	0.395 (0.293–0.503)	0.358 (0.245–0.481)	n.a.	n.a.
Mirtazapine	0.423 (0.371–0.476)	0.434 (0.374–0.496)	0.458 (0.356–0.565)	0.470 (0.212–0.741)
Nortriptyline	0.441 (0.275–0.617)	0.450 (0.284–0.628)	0.455 (0.287–0.633)	n.a.
Paroxetine	0.410 (0.374–0.445)	0.416 (0.377–0.455)	0.414 (0.370–0.459)	0.443 (0.388–0.499)
Reboxetine	0.404 (0.252–0.566)	0.399 (0.252–0.561)	n.a.	n.a.
Sertraline	0.400 (0.359–0.443)	0.409 (0.366–0.453)	0.418 (0.358–0.479)	0.383 (0.193–0.594)
Venlafaxine	0.433 (0.401–0.465)	0.439 (0.404–0.474)	0.451 (0.409–0.493)	0.499 (0.439–0.560)

N.a  =  not available. 95% credibility intervals in brackets.

### Cost-effectiveness

The results of the cost-effectiveness analysis are shown in Table 4. Despite its relatively high acquisition cost, the SSRI escitalopram is associated with a lower total cost compared with all other treatment strategies, reflecting the fact that patients on average spend less time in the costly depression state. Furthermore, escitalopram is associated with a larger health gain (QALYs) at one year, and therefore *dominates* the other treatment strategies as more QALYs are achieved at a lower total cost.

**Table 4:** Results cost-effectiveness analysis with societal perspective.

Drug	Cost (€)	QALY	ICER
**Escitalopram**	14 755(12 646–17 086)	0.6978(0.6512–0.7411)	base
**Venlafaxine**	14 878(12 713–17 268)	0.6942(0.6477–0.7378)	dom
**Duloxetine**	15 082(12 886–17 496)	0.6933(0.6463–0.7372)	dom
**Mirtazapine**	14 961(12 756–17 428)	0.6926(0.6457–0.7368)	dom
**Paroxetine**	15 080(12 857–17 545)	0.6906(0.6438–0.7348)	dom
**Sertraline**	15 159(12 908–17 657)	0.6892(0.6422–0.7333)	dom
**Citalopram**	15 343(13 033–17 906)	0.6861(0.6386–0.7308)	dom
**Fluoxetine**	15 428(13 104–17 998)	0.6847(0.6373–0.7293)	dom

QALY  =  quality-adjusted life year; ICER  =  incremental cost-effectiveness ratio; base  =  cheapest alternative; dom  =  dominated. Results are shown as means and 95% credibility intervals.

### Alternative Scenarios

When only the direct health care costs are included, all treatments but venlafaxine and escitalopram are dominated. Escitalopram is associated with marginally higher costs (€14) and better outcomes (0.004 QALYs) compared with venlafaxine yielding a cost per QALY of €3,732 (Table 5). Alternative scenarios such as using efficacy data from all study durations or varying the probabilities of relapse or suicide rates did not alter the results of the analysis. Results are available from the authors on request.

**Table 5:** Results cost-effectiveness analysis with health-care perspective.

Drug	Cost	QALY	ICER
**Escitalopram**	5 088(4 250–6 054)	0.6978(0.6512–0.7411)	ICER 3 732 to venlafaxine
**Venlafaxine**	5 074(4 217–6 072)	0.6942(0.6477–0.7378)	base
**Duloxetine**	5 247(4 383–6 247)	0.6933(0.6463–0.7372)	dom
**Mirtazapine**	5 099(4 225–6 117)	0.6926(0.6457–0.7368)	dom
**Paroxetine**	5 143(4 262–6 166)	0.6906(0.6438–0.7348)	dom
**Sertraline**	5 167(4 276–6 202)	0.6892(0.6422–0.7333)	dom
**Citalopram**	5 235(4 323–6 297)	0.6861(0.6386–0.7308)	dom
**Fluoxetine**	5 267(4 350–6 331)	0.6847(0.6373–0.7293)	dom

QALY  =  quality-adjusted life year; ICER  =  incremental cost-effectiveness ratio; base  =  cheapest alternative; dom  =  dominated. Means and 95% credibility intervals.

### Probabilistic Analysis

**Figure 4:**
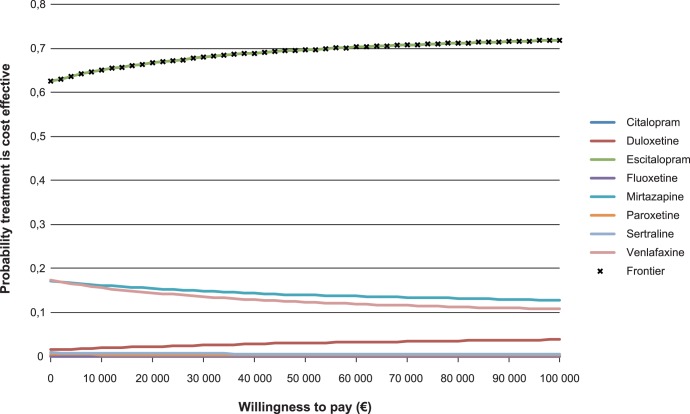
Cost-effectiveness acceptability curves. Note: All treatments except escitalopram, mirtazapine, venlafaxine and duloxetine have a low probability of being cost effective at all willingness-to-pay values of a health outcome, hence the curves for these treatments virtually lie on the abscissa.

The cost-effectiveness acceptability curves are presented in Figure 4. For every value of willingness to pay, or threshold value for cost-effectiveness, there is a high probability that escitalopram is cost-effective.

## Discussion

Our network meta-analysis supports the conclusion from previous reviews that, in certain populations, the newest antidepressants have a somewhat better efficacy than the generically available SSRIs. In our decision-analytic model we found that using escitalopram instead of other antidepressants was associated with a reduction in time spent in depression, leading to marginally more QALYs at no additional cost.

As noted in Table 4, there are large amounts of uncertainty in the total costs and QALYs for each investigated treatment and the differences in effect between some of the investigated treatments are rather small. Still, the probability that escitalopram is cost-effective is approximately 70% at a wide range of willingness-to-pay thresholds (Figure 4). A key finding of the present study is that the relatively small differences in remission rates between therapies may have relatively large implications for differences in costs, in particular from a societal perspective, whereas the corresponding differences in QALYs are rather small.

Remission of symptoms is nowadays seen as a better outcome to study than response (defined e.g. as a 50% decrease on a rating scale). However, our analysis is generally in strong agreement with a previous study using response as outcome [8]. This lends some support to the fact that the choice of endpoint to measure efficacy may not be crucial. However, the present study which explicitly attempted to estimate costs and quality of life implications of remission indicates that escitalopram is cost-effective. This conclusion is different from the tentative conclusion that sertraline is the most cost-effective alternative reported in a previous study [8].

An interesting observation was that the odds ratio for escitalopram against citalopram is lower in the MTC compared with pooling the head to head studies, which were all sponsored by the manufacturer of these drugs. We note that in several cases, the odds ratios from pooling direct comparative studies differ from those reported in the multiple treatment comparison. This is not surprising as the latter includes a larger body of evidence, but the underlying reasons for the differences could be worthy of further investigation as this may partly reflect a “wish-effect” (or sponsor bias) in the trials. This potential bias is reduced in the multiple treatment comparison where the drugs also appear as non-sponsored comparators.

Our study has some important strengths. First, it provides a thorough investigation regarding probabilities of remission in major depression. The large number of studies included in the systematic review provides a solid evidence base for the meta-analysis, and by using Bayesian multiple treatment comparison techniques all available evidence is efficiently utilised in the evidence synthesis. Second, against the background of little previous research evaluating the cost-effectiveness of all relevant antidepressants simultaneously in a primary care setting, we provide a framework for their evaluation. The study provides a first attempt to estimate health outcomes and cost consequences of achieving remission and as such also supports health-care decision makers in their need to include cost-effectiveness considerations systematically when deciding how to best allocate scarce health-care resources in the treatment of major depressive disorder.

Our study also has limitations. The cost data used in the health economic model come from Sweden, which could reduce generalizability of the results to other countries and settings. However, there is little evidence that the costs would differ dramatically in other European countries [7] and the results are also quite robust to changes in the cost parameters. Second, studies with six weeks duration or more were included in the meta-analysis, but six weeks may be too short to study remission. However, excluding studies of duration less than eight weeks did not alter the results significantly. A related limitation concerns selection bias regarding which studies were included in the meta-analysis. A genuine attempt was made to locate unpublished material, but we may of course have failed to find important studies. Also, studies were only included if remission was reported. It has been suggested that this endpoint is more likely to be reported if a positive effect is shown for the sponsor’s drug [29]. A solution to this would be to calculate remission for all studies in uniform fashion as is done in Omori et al [30]. Furthermore the present study is limited to pharmacologic therapy and it should be recognized that both psychological and pharmacologic therapies appear effective in the treatment of depressive disorders, each with its own merits. It is very important to be able to individualize treatment of depression given the idiosyncratic response and tolerability issues as well as the lack of patient adherence. Our multiple treatment comparison is only able to address a subset of this large issue. However, for many decision makers, the question of whether there are efficacy (or cost-effectiveness) differences between antidepressant drugs is in itself important and worthy of careful analysis. An area for further research would be to incorporate psychological and pharmacologic therapies in a joint evaluation. Such an analysis could be based on the methods outlined in this work, although the approach to the evidence synthesis and cost-effectiveness model would have to be slightly amended.

How side effects should be handled in the analysis is a difficult question. Side effects are reported in a much less consistent way than efficacy and the reporting itself thus introduces a bias into the analysis. The most reliable measure is probably *drop outs due to adverse events*. This is also a clinically relevant measure which directly affects the efficacy of the treatment. It should be noted that drop-out effects on efficacy are captured in the efficacy measure in the trials and is therefore included in the cost-effectiveness evaluation. However, there is also a direct effect on patient’s quality of life from negative side effects. The QALY-weights used in the current cost-effectiveness evaluation include side effects, but represent only an average negative effect since they were measured in a naturalistic setting and the treatments of individual patients were not reported. It should be noted that escitalopram had a rather favorable side-effect profile compared to the other treatments (Table S3) and it is unlikely that the results would have changed if the effect of adverse events on quality of life had been explicitly modeled. Rather, we would expect the present results to be further reinforced should such an approach be taken.

Finally, it should be noted that the MTC only included active therapy and placebo arms were excluded. While we recognize that this design actually leave out some relevant evidence as placebo controlled trials also include important information on active therapy, head to head trials are the gold standard in generating clinical efficacy data and including only head to head trials implies that a potential placebo effect is actually removed from the measurement of relative efficacy in the meta-analysis.

### Conclusion

Employing a large body of randomized head-to-head evidence, escitalopram has the highest probability of remission of the investigated antidepressants and is the most effective and cost-effective pharmacological treatment strategy for moderate to severe depression in a primary care setting, when evaluated over a one year time-horizon. The difference in effect is modest but even small differences in remission rates may be important when assessing costs and cost-effectiveness of antidepressants.

## Supporting Information

Table S1 **Detailed description of model parameters.**
(DOCX)

Table S2 **Summary of studies included in meta-analysis.**
(DOC)

Table S3 **Frequency of drop outs due to adverse events.**
(DOCX)
