# Supplementary material for: Effectiveness and Cost-Effectiveness of Antidepressants in Primary Care: A Multiple Treatment Comparison Meta-Analysis and Cost-Effectiveness Model
Source: PLoS One. 2012 Aug 2;7(8):e42003. doi: 10.1371/journal.pone.0042003 (PMC3410906; doi:10.1371/journal.pone.0042003)
Supplement: Table S1 — Detailed description of model parameters. (DOCX) [file pone.0042003.s001.docx]

| **Parameter** | **Mean** | | **Distribution** | **Comment** | **Source** |
| --- | --- | --- | --- | --- | --- |
| Healthcare costs in depression in primary care (per month) | € 374 | | Gamma(91.96, 0.2459) | Cost derived from the reported 6-month results. Gamma distribution derived from the reported mean and confidence interval. | Sobocki P, Ekman M, Agren H, Runeson B, Jonsson B (2006) The mission is remission: health economic consequences of achieving full remission with antidepressant treatment for depression. Int J Clin Pract 60: 791-798 |
| Healthcare costs in remission in primary care (per month) | € 273 | | Gamma(105.29, 0.4461) | Cost derived from the reported 6-month results. Gamma distribution derived from the reported mean and confidence interval. | As above |
| Healthcare costs in depression in specialist care (per month) | € 748 | | Normal(2, 0.25) | Based on the modelling in Löthgren, the costs in specialist care were estimated to be twice as high compared to primary care. Uncertainty in this estimate was incorporated as a Normal distribution around the estimated increase in costs compared with primary care. | Löthgren M, Hemels M, François C, Jönsson B (2004) A cost-effectiveness analysis of escitalopram as first line treatment of depression in Sweden. Primary Care Psychiatry 9: 153-162 |
| Healthcare costs in remission in specialist care (per month) | € 546 | | Normal(2, 0.25) | Based on the modelling in Löthgren, the costs in specialist care were estimated to be twice as high compared to primary care. Uncertainty in this estimate was incorporated as a Normal distribution around the estimated increase in costs compared with primary care. | As above |
| Societal (healthcare and non-healthcare) cost in depression in primary care (per month) | € 1 185 | | Gamma(208.84, 0.1762) | Cost derived from the reported 6-month results. Gamma distribution derived from the reported mean and confidence interval. | Sobocki P, Ekman M, Agren H, Runeson B, Jonsson B (2006) The mission is remission: health economic consequences of achieving full remission with antidepressant treatment for depression. Int J Clin Pract 60: 791-798 |
| Societal (healthcare and non-healthcare) cost in remission in primary care (per month) | € 715 | | Gamma(151.97, 0.2125) | Cost derived from the reported 6-month results. Gamma distribution derived from the reported mean and confidence interval. | As above |
| Societal (healthcare and non-healthcare) cost in depression in specialist care (per month) | € 2 370 | | Normal(2, 0.25) | Based on the modelling in Löthgren, the costs in specialist care were estimated to be twice as high compared to primary care. Uncertainty in this estimate was incorporated as a Normal distribution around the estimated increase in costs compared with primary care. | Löthgren M, Hemels M, François C, Jönsson B (2004) A cost-effectiveness analysis of escitalopram as first line treatment of depression in Sweden. Primary Care Psychiatry 9: 153-162 |
| Societal (healthcare and non-healthcare) cost in remission in specialist care (per month) | € 1 430 | | Normal(2, 0.25) | Based on the modelling in Löthgren, the costs in specialist care were estimated to be twice as high compared to primary care. Uncertainty in this estimate was incorporated as a Normal distribution around the estimated increase in costs compared with primary care. | As above |
| Loss of production deceased (per month) | € 3 548 | | Deterministic | Monthly average wage cost according to Statistics Sweden. | Statistics Sweden (www.scb.se) |
| Cost of dying from suicide (one-off cost) | € 3 495 | | Gamma(12.22, 0.0035) | Estimated direct cost of suicide. A standard error of 1000 was assumed as no uncertainty estimates were reported. | [Räddningsverket (2004) Suicid och samhällsekonomiska kostnader. NCO 2004:7 (https://www.msb.se/Upload/Kunskapsbank/Statistik_larande/Suicid_och_samhallsekonomiska_kostnader.pdf)](https://www.msb.se/Upload/Kunskapsbank/Statistik_larande/Suicid_och_samhallsekonomiska_kostnader.pdf) |
| Cost of treatment switch (one-off cost) | € 150 | | Deterministic | Cost of physician visit according to price list at the Academic Hospital, Uppsala, Sweden. | http://www.externt9.lul.se/svn/index.htm |
| Cost of suicide attempt (one-off cost) | € 11 753 | | Gamma(5.52, 0.0005) | Estimated direct cost of suicide attempt. A standard error of 5000 was assumed as no uncertainty estimates were reported. | [Räddningsverket (2004) Suicid och samhällsekonomiska kostnader, NCO 2004:7. (https://www.msb.se/Upload/Kunskapsbank/Statistik_larande/Suicid_och_samhallsekonomiska_kostnader.pdf)](https://www.msb.se/Upload/Kunskapsbank/Statistik_larande/Suicid_och_samhallsekonomiska_kostnader.pdf) |
| QALY weight remission | 0.810 | | Beta(311.65, 72.10) | Standard error (0.02) derived from reported confidence interval. | Sobocki P, Ekman M, Agren H, Runeson B, Jonsson B (2006) The mission is remission: health economic consequences of achieving full remission with antidepressant treatment for depression. Int J Clin Pract 60: 791-798 |
| Decrement in utility in depression | 0.240 | | Gamma(144, 600) | Standard error (0.02) derived from reported confidence interval. | As above |
| Probability remission after switch | 0.248 | | Beta(24.8, 75.2) | Assumed distribution due to lack of uncertainty information. A wide distribution assuming a sample of 100 patients was applied. | STAR D investigators. *Rush AJ, Trivedi MH, Wisniewski SR, Stewart JW, Nierenberg AA, et al. (2006) Bupropion-SR, sertraline, or venlafaxine-XR after failure of SSRIs for depression. N Engl J Med 354: 1231-1242. |
| Probability relapse | 0.110 | | Beta(11, 89) | Assumed distribution due to lack of uncertainty information. A wide distribution assuming a sample of 100 patients was applied. | Gilchrist G, Gunn J (2007) Observational studies of depression in primary care: what do we know? BMC Fam Pract 8: 28. |
| Probability suicide attempt in depression | 0.031 | | Beta(3.1, 96.9) | Assumed distribution due to lack of uncertainty information. A wide distribution assuming a sample of 100 patients was applied. | Khan A, Khan SR, Leventhal RM, Brown WA (2001) Symptom reduction and suicide risk in patients treated with placebo in antidepressant clinical trials: a replication analysis of the Food and Drug Administration Database. Int J Neuropsychopharmacol 4: 113-118. |
| Probability dying of suicide attempt | 0.110 | | Beta(11, 89) | Assumed distribution due to lack of uncertainty information. A wide distribution assuming a sample of 100 patients was applied. | [Centers for Disease Control and Prevention, National Center for Injury Prevention and Control. Web-based Injury Statistics Query and Reporting System (WISQARS): (www.cdc.gov/ncipc/wisqars)](http://www.cdc.gov/ncipc/wisqars) |
| Probability remission after specialist care | | 0.248 | Beta(24.8, 75.2) | Assumed to be same as remission after switch. |  |
