# Supplementary material for: Effectiveness and Cost-Effectiveness of Antidepressants in Primary Care: A Multiple Treatment Comparison Meta-Analysis and Cost-Effectiveness Model
Source: PLoS One. 2012 Aug 2;7(8):e42003. doi: 10.1371/journal.pone.0042003 (PMC3410906; doi:10.1371/journal.pone.0042003)
Supplement: Table S2 — Summary of studies included in meta-analysis. (DOC) [file pone.0042003.s002.doc]

| **Study** | **Reference** | **Drug 1** | **Drug 2** | **Setting** | **Duration**  **in weeks** | **N drug 1*** | **Remission drug 1**** | **N drug 2*** | **Remission drug 2**** | **Sponsor** |
| --- | --- | --- | --- | --- | --- | --- | --- | --- | --- | --- |
| Schatzberg et al 2006 |  | Flouxetine | Venlafaxine | Outpatient | 8 | 99 | 20 | 93 | 25 | Wyeth |
| Rudolph & Feiger 1999 |  | Flouxetine | Venlafaxine | Outpatient | 8 | 103 | 23 | 95 | 35 | Wyeth |
| Rudolph & Feiger 1997 |  | Fluoxetine | Venlafaxine | Outpatient | 12 | 170 | 58 | 171 | 60 | GSK |
| Alves et al 1999 |  | Fluoxetine | Venlafaxine | Outpatient | 12 | 47 | 19 | 40 | 20 | Pfizer |
| Nemeroff & Thase 2007 |  | Fluoxetine | Venlafaxine | Outpatient | 6 | 101 | 28 | 96 | 31 | Wyeth |
| De Nayer et al 2002 |  | Fluoxetine | Venlafaxine | Outpatient | 12 | 67 | 27 | 64 | 38 | Wyeth |
| Costa e Silva 1998 |  | Fluoxetine | Venlafaxine | Outpatient | 8 | 186 | 112 | 196 | 118 | Wyeth |
| Kornaat 1998 |  | Fluoxetine | Venlafaxine | Outpatient | 8 | 77 | 14 | 79 | 19 | Whyet |
| S332 | Unpublished | Flouxetine | Venlafaxine | Outpatient | 6 | 23 | 10 | 24 | 9 | n.r. |
| Rudolph et al 1998 |  | Flouxetine | Venlafaxine | Outpatient | 6 | 146 | 49 | 144 | 62 | n.r. |
| S606 | Unpublished | Flouxetine | Venlafaxine | Outpatient | 6 | 63 | 37 | 64 | 44 | n.r. |
| S102 | Unpublished | Flouxetine | Venlafaxine | Outpatient | 8 | 26 | 12 | 28 | 14 | n.r. |
| Dierick et al 1996 |  | Flouxetine | Venlafaxine | Outpatient | 8 | 157 | 71 | 145 | 75 | Wyeth |
| Keller et al 2007 |  | Flouxetine | Venlafaxine | Outpatient | 10 | 266 | 132 | 781 | 380 | n.r. |
| Mehtonen 2002 |  | Flouxetine | Venlafaxine | Outpatient | 10 | 50 | 28 | 50 | 30 | Wyeth |
| Silverstone et al 1999 |  | Flouxetine | Venlafaxine | Outpatient | 12 | 119 | 33 | 122 | 37 | Wyeth |
| Stevens 1997 |  | Flouxetine | Venlafaxine | Outpatient | 12 | 114 | 26 | 102 | 26 | Wyeth |
| Tzanakaki et al 2000 |  | Fluoxetine | Venlafaxine | Inpatient | 6 | 54 | 19 | 55 | 22 | Wyeth |
| Cantillon & Daley 2000 |  | Flouxetine | Venlafaxine | Inpatient | 6 | 99 | 17 | 91 | 24 | n.r. |
| Clerc et al 1994 |  | Flouxetine | Venlafaxine | Inpatient | 6 | 34 | 9 | 33 | 19 | Wyeth |
| Gagiano 1993 |  | Fluoxetine | Paroxetine | Outpatient | 6 | 45 | 28 | 45 | 26 | GSK |
| Tignol 1993 |  | Fluoxetine | Paroxetine | Inpatient | 6 | 87 | 42 | 89 | 47 | GSK |
| Geretsegger et al 1994 |  | Fluoxetine | Paroxetine | Both | 6 | 52 | 9 | 54 | 11 | GSK |
| deWilde et al 1993 |  | Fluoxetine | Paroxetine | n.r. | 6 | 50 | 19 | 49 | 22 | GSK |
| Hong et al 2003 |  | Fluoxetine | Mirtazapine | Outpatient | 6 | 59 | 16 | 60 | 21 | Organon |
| Versiani et al 2005 |  | Fluoxetine | Mirtazapine | Inpatient | 8 | 147 | 61 | 145 | 58 | Organon |
| Amini et al 2005 |  | Fluoxetine | Mirtazapine | Both | 6 | 18 | 4 | 18 | 7 | n.r. |
| Wheatley et al 1998 |  | Fluoxetine | Mirtazapine | Both | 6 | 63 | 16 | 60 | 14 | Organon |
| Kasper et al 2005 |  | Fluoxetine | Escitalopram | Both | 8 | 164 | 49 | 170 | 68 | Wyeth |
| Goldstein et al 2002 |  | Fluoxetine | Duloxetine | Outpatient | 8 | 33 | 10 | 66 | 28 | Lilly |
| Beasley et al 1993 |  | Flouxetine | Imipramine | Inpatient | 6 | 56 | 12 | 62 | 21 | Lilly |
| Patris et al 1996 |  | Flouxetine | Citalopram | Outpatient | 8 | 184 | 109 | 173 | 115 | Lundbeck |
| Ballus et al 2000 |  | Venlafaxine | Paroxetine | Outpatient | 12 | 41 | 23 | 43 | 14 | Wyeth |
| McPartlin et al 1998 |  | Venlafaxine | Paroxetine | Outpatient | 12 | 183 | 98 | 178 | 93 | Whyet |
| S349 | Unpublished | Venlafaxine | Paroxetine | Outpatient | 8 | 75 | 25 | 80 | 26 | n.r. |
| Casabona et al 2002 |  | Venlafaxine | Paroxetine | Outpatient | 8 | 57 | 18 | 52 | 20 | Wyeth |
| Salinas 1997 |  | Venlafaxine | Paroxetine | Outpatient | 8 | 161 | 82 | 80 | 30 | Wyeth |
| Dufour et al 2001 |  | Venlafaxine | Paroxetine | Outpatient | 12 | 173 | 78 | 180 | 76 | Lundbeck |
| S632 | Unpublished | Venlafaxine | Paroxetine | Inpatient | 8 | 40 | 18 | 45 | 18 | Lundbeck |
| Shelton et al 2006 |  | Venlafaxine | Sertraline | Outpatient | 8 | 76 | 37 | 82 | 31 | Pfizer |
| Sir et al 2005 |  | Venlafaxine | Sertraline | Outpatient | 8 | 79 | 43 | 79 | 47 | Pfizer |
| Mehtonen et al 2000 |  | Venlafaxine | Sertraline | Outpatient | 8 | 75 | 40 | 72 | 27 | Wyeth |
| S402 | Unpublished | Venlafaxine | Sertraline | Inpatient | 10 | 287 | 116 | 288 | 96 | Wyeth |
| S414 | Unpublished | Venlafaxine | Sertraline | Inpatient | 10 | 288 | 89 | 294 | 95 | Wyeth |
| Bielski et al 2004 |  | Venlafaxine | Escitalopram | Outpatient | 8 | 100 | 31 | 98 | 35 | Forest |
| Montgomery et al 2004 |  | Venlafaxine | Escitalopram | Outpatient | 8 | 142 | 99 | 146 | 102 | n.r. |
| Lenox-Smith et al 2001 |  | Venlafaxine | Citalopram | Inpatient | 12 | 193 | 66 | 198 | 56 | n.r. |
| Guelfi et al 2001 |  | Venlafaxine | Mirtazapine | Inpatient | 8 | 75 | 21 | 77 | 29 | n.r. |
| Gentil et al 2000 |  | Venlafaxine | Amitriptyline | Outpatient | 8 | 57 | 33 | 59 | 32 | Wyeth |
| Perahia et al 2008 |  | Venlafaxine | Duloxetine | Outpatient | 6 | 330 | 116 | 318 | 100 | Lilly |
| Hacket et al 1998 |  | Venlafaxine | Fluvoxamine | inpatient | 6 | 77 | 38 | 34 | 12 | n.r. |
| HMAT study group A | Unpublished | Paroxetine | Duloxetine | Outpatient | 8 | 87 | 31 | 81 | 23 | Lilly |
| Perahia et al 2006 |  | Paroxetine | Duloxetine | Outpatient | 8 | 97 | 42 | 93 | 41 | Lilly |
| Goldstein et al 2004 |  | Paroxetine | Duloxetine | Outpatient | 8 | 84 | 31 | 86 | 43 | Lilly |
| Detke et al 2004 |  | Paroxetine | Duloxetine | Outpatient | 8 | 85 | 37 | 93 | 47 | Lilly |
| Lee et al 2007 |  | Paroxetine | Duloxetine | n.r. | 8 | 240 | 121 | 238 | 117 | n.r. |
| Benkert et al 2000 |  | Paroxetine | Mirtazapine | Outpatient | 6 | 123 | 42 | 127 | 52 | Organon |
| Schatzberg et al 2002 |  | Paroxetine | Mirtazapine | Outpatient | 8 | 120 | 34 | 126 | 48 | Organon |
| Wade et al 2003 |  | Paroxetine | Mirtazapine | Outpatient | 8 | 84 | 21 | 93 | 25 | Organon |
| Boulenger et al 2006 |  | Paroxetine | Escitalopram | Outpatient | 12 | 223 | 114 | 228 | 143 | Wyeth |
| Baldwin et al 2006 |  | Paroxetine | Escitalopram | Both | 8 | 156 | 96 | 165 | 93 | Organon |
| Arminen et al 1994 |  | Paroxetine | Imipramine | Inpatient | 12 | 25 | 11 | 32 | 12 | Organon |
| 29060/056/uk | Unpublished | Paroxetine | Dothiepin | n.r. | 6 | 59 | 33 | 62 | 32 | GSK |
| Moon & Vince 1996 |  | Paroxetine | Lofepramine | Outpatient | 6 | 60 | 33 | 62 | 32 | GSK |
| SER-CHN-1 | Unpublished | Paroxetine | Amitriptyline | n.r. | 6 | 113 | 54 | 118 | 40 | GSK |
| Åberg-Wistedt et al 2000 |  | Paroxetine | Sertraline | Outpatient | 8 | 177 | 101 | 176 | 91 | Pfizer |
| Yoshimura et al 2007 |  | Paroxetine | Milnacipran | Both | 8 | 21 | 12 | 21 | 10 | Wyeth |
| Laurelle et al 1991 |  | Paroxetine | Maprotilin | Inpatient | 6 | 28 | 10 | 32 | 11 | GSK |
| Nierenberg et al 2007 |  | Escitalopram | Duloxetine | Outpatient | 8 | 274 | 88 | 273 | 101 | Lilly |
| Jonas et al 2006 |  | Escitalopram | Duloxetine | Outpatient | 8 | 136 | 54 | 126 | 45 | Forest |
| Wade et al 2007 |  | Escitalopram | Duloxetine | Outpatient | 8 | 141 | 74 | 146 | 70 | Lundbeck |
| Khan et al 2007 |  | Escitalopram | Duloxetine | Outpatient | 8 | 136 | 56 | 126 | 44 | Organon |
| Colonna et al 2005 |  | Escitalopram | Citalopram | Outpatient | 8 | 165 | 91 | 174 | 78 | Lundbeck |
| Moore et al 2005 |  | Escitalopram | Citalopram | Outpatient | 8 | 138 | 75 | 142 | 61 | Lundbeck |
| Lepola et al 2003 |  | Escitalopram | Citalopram | Outpatient | 8 | 155 | 81 | 159 | 68 | Lundbeck |
| Ventura et al 2007 |  | Escitalopram | Sertraline | Outpatient | 8 | 104 | 51 | 107 | 57 | Forest |
| van Amerongen et al 2002 |  | Milnicipran | Imipramine | Inpatient | 6 | 53 | 30 | 56 | 29 | Pierre Fabre |
| Tignol et al 1998 |  | Milnicipran | Imipramine | Both | 8 | 112 | 36 | 107 | 38 | Lundbeck |
| Behnke et al 2003 |  | Mirtazapine | Sertraline | Both | 8 | 171 | 76 | 168 | 73 | Pierre Fabre |
| Rossini et al 2005 |  | Sertraline | Fluvoxamine | Inpatient | 7 | 48 | 25 | 40 | 28 | n.r. |
| Wisner et al 2006 |  | Sertraline | Nortriptylin | Outpatient | 8 | 55 | 25 | 54 | 26 | Pfizer |
| Keller et al 1998 |  | Sertraline | Imipramine | Outpatient | 12 | 426 | 175 | 209 | 82 | Wyeth |
| Haffmans et al 1996 |  | Citalopram | Fluvoxamine | Both | 6 | 108 | 15 | 109 | 9 | Lundbeck |
| Kyle et al 1998 |  | Citalopram | Amitriptyline | Outpatient | 8 | 179 | 96 | 186 | 99 | Lundbeck |
| Birkenhäger et al 2004 |  | Fluvoxamine | Imipramine | Inpatient | 6 | 68 | 10 | 70 | 16 | Solvay |
| Schwartz et al 2002 |  | Reboxetine | Venlafaxin | n.r. | 8 | 80 | 20 | 87 | 21 | n.r. |
| Blier et al 2009 |  | Mirtazapine | Paroxetine | Both | 6 | 21 | 4 | 19 | 5 | Organon |

*Number of patients evaluated for remission. **Number of patients achieving remission. n.r. = not reported.

References

1. Schatzberg A, Roose S (2006) A double-blind, placebo-controlled study of venlafaxine and fluoxetine in geriatric outpatients with major depression. Am J Geriatr Psychiatry 14: 361-370.

2. Rudolph RL, Feiger AD (1999) A double-blind, randomized, placebo-controlled trial of once-daily venlafaxine extended release (XR) and fluoxetine for the treatment of depression. J Affect Disord 56: 171-181.

3. Rudolph RL, Feiger AD (1997) A double-blind, randomized, placebo-controlled trial of once-daily venlafaxine extended release (XR) and fluoxetine for the treatment of depression. Primary Care Psychiatry 3: 51-58.

4. Alves C, Cachola I, Brandao J (1999) Efficacy and tolerability of venlafaxine and fluoxetine in outpatients with major depression. Primary Care Psychiatry 5: 57-63.

5. Nemeroff CB, Thase ME (2007) A double-blind, placebo-controlled comparison of venlafaxine and fluoxetine treatment in depressed outpatients. J Psychiatr Res 41: 351-359.

6. De Nayer A, Geerts S, Ruelens L, Schittecatte M, De Bleeker E, et al. (2002) Venlafaxine compared with fluoxetine in outpatients with depression and concomitant anxiety. Int J Neuropsychopharmacol 5: 115-120.

7. Costa e Silva J (1998) Randomized, double-blind comparison of venlafaxine and fluoxetine in outpatients with major depression. J Clin Psychiatry 59: 352-357.

8. Koornat H. Randomized, double-blind comparison of venlafaxine and fluoxetine for moderately depressed outpatients; 1998; Glasgow, Scotland.

9. Rudolph RL, Entsuah R, Aguiar L, Derivan A (1998) Early onset of antidepressant activity of venlafaxine compared with placebo and fluoxetine in outpatients in a double-blind study. The Journal of the European College of Neuropsychopharmacology 8 (suppl 2): S142.

10. Dierick M, Ravizza L, Realini R, Martin A (1996) A double-blind comparison of venlafaxine and fluoxetine for treatment of major depression in outpatients. Prog Neuropsychopharmacol Biol Psychiatry 20: 57-71.

11. Keller MB, Trivedi MH, Thase ME, Shelton RC, Kornstein SG, et al. (2007) The Prevention of Recurrent Episodes of Depression with Venlafaxine for Two Years (PREVENT) Study: Outcomes from the 2-year and combined maintenance phases. J Clin Psychiatry 68: 1246-1256.

12. Mehtonen O-P (2002) A double-blind, randomized study of the efficacy and safety of venlafaxine extended release (ER) versus fluoxetine in outpatients with major depression. The Journal of the European College of Neuropsychopharmacology 12 (suppl 3): 253.

13. Silverstone PH, Ravindran A (1999) Once-daily venlafaxine extended release (XR) compared with fluoxetine in outpatients with depression and anxiety. Venlafaxine XR 360 Study Group. J Clin Psychiatry 60: 22-28.

14. Stevens I (1997) Comparison of the efficacy and safety of venlafaxine and fluoxetine in GP patients with moderate to severe depression. Biol Psychiatry, 1997 42 (suppl): 244S.

15. Tzanakaki M, Guazzelli M, Nimatoudis I, Zissis NP, Smeraldi E, et al. (2000) Increased remission rates with venlafaxine compared with fluoxetine in hospitalized patients with major depression and melancholia. Int Clin Psychopharmacol 15: 29-34.

16. Cantillon, Daley (2000) Poster. Forum on Mood and Anxiety Disorders. Monte Carlo, Monaco.

17. Clerc GE, Ruimy P, Verdeau-Palles J (1994) A double-blind comparison of venlafaxine and fluoxetine in patients hospitalized for major depression and melancholia. The Venlafaxine French Inpatient Study Group. Int Clin Psychopharmacol 9: 139-143.

18. Gagiano C (1993) A double-blind comparison of paroxetine and fluoxetine in patients with major depression. Br J Clin Res 4: 145-152.

19. Tignol J (1993) A double-blind, randomized, fluoxetine-controlled, multicenter study of paroxetine in the treatment of depression. J Clin Psychopharmacol 13: 18S-22S.

20. Geretsegger C, Bohmer F, Ludwig M (1994) Paroxetine in the elderly depressed patient: randomized comparison with fluoxetine of efficacy, cognitive and behavioural effects. Int Clin Psychopharmacol 9: 25-29.

21. De Wilde J, Spiers R, Mertens C, Bartholome F, Schotte G, et al. (1993) A double-blind, comparative, multicentre study comparing paroxetine with fluoxetine in depressed patients. Acta Psychiatr Scand 87: 141-145.

22. Hong CJ, Hu WH, Chen CC, Hsiao CC, Tsai SJ, et al. (2003) A double-blind, randomized, group-comparative study of the tolerability and efficacy of 6 weeks' treatment with mirtazapine or fluoxetine in depressed Chinese patients. J Clin Psychiatry 64: 921-926.

23. Versiani M, Moreno R, Ramakers-van Moorsel CJ, Schutte AJ (2005) Comparison of the effects of mirtazapine and fluoxetine in severely depressed patients. CNS Drugs 19: 137-146.

24. Amini H, Aghayan S, Jalili S, Akhondzadeh S, Yahyazadeh O, et al. (2005) Comparison of mirtazapine and fluoxetine in the treatment of major depressive disorder: a double-blind, randomized trial. J Clin Pharm Ther 30: 133-138.

25. Wheatley DP, van Moffaert M, Timmerman L, Kremer CM (1998) Mirtazapine: efficacy and tolerability in comparison with fluoxetine in patients with moderate to severe major depressive disorder. Mirtazapine-Fluoxetine Study Group. J Clin Psychiatry 59: 306-312.

26. Kasper S, de Swart H, Friis Andersen H (2005) Escitalopram in the treatment of depressed elderly patients. Am J Geriatr Psychiatry 13: 884-891.

27. Goldstein DJ, Mallinckrodt C, Lu Y, Demitrack MA (2002) Duloxetine in the treatment of major depressive disorder: a double-blind clinical trial. J Clin Psychiatry 63: 225-231.

28. Beasley CM, Jr., Holman SL, Potvin JH (1993) Fluoxetine compared with imipramine in the treatment of inpatient depression. A multicenter trial. Ann Clin Psychiatry 5: 199-207.

29. Patris M, Bouchard JM, Bougerol T, Charbonnier JF, Chevalier JF, et al. (1996) Citalopram versus fluoxetine: a double-blind, controlled, multicentre, phase III trial in patients with unipolar major depression treated in general practice. Int Clin Psychopharmacol 11: 129-136.

30. Ballus C, Quiros G, De Flores T, de la Torre J, Palao D, et al. (2000) The efficacy and tolerability of venlafaxine and paroxetine in outpatients with depressive disorder or dysthymia. Int Clin Psychopharmacol 15: 43-48.

31. McPartlin G, Reynolds A, Anderson C, Casoy J (1998) A comparison of once-daily venlafaxine XR and paroxetine in depressed outpatients treated in general practice. Primary Care Psychiatry 4: 127-132.

32. Casabona GM, Silenzi V, Guazzelli M (2002) A randomized, double blind, comparison of venlafaxine ER and paroxetine in outpatients with moderate to severe major depression. The Journal of the European College of Neuropsychopharmacology 12, supplement 3: 208.

33. Salinas E (1997) Once-daily extended release (XR) venlafaxine versus paroxetine in outpatients with major depression. Biol Psychiatry 42 (suppl): 244S.

34. Dufour A, Van Hauteghem D, Slachmuylders P, Leyman S, Mignon A (2001) Clinical acceptability of venlafaxine extended release and paroxetine in outpatients treated for depression by general practioners. J Europ Coll Neuropsycho-pharm 2001;11 (suppl 3): 11 (suppl 3): 224.

35. Shelton RC, Haman KL, Rapaport MH, Kiev A, Smith WT, et al. (2006) A randomized, double-blind, active-control study of sertraline versus venlafaxine XR in major depressive disorder. J Clin Psychiatry 67: 1674-1681.

36. Sir A, D'Souza RF, Uguz S, George T, Vahip S, et al. (2005) Randomized trial of sertraline versus venlafaxine XR in major depression: efficacy and discontinuation symptoms. J Clin Psychiatry 66: 1312-1320.

37. Mehtonen OP, Sogaard J, Roponen P, Behnke K (2000) Randomized, double-blind comparison of venlafaxine and sertraline in outpatients with major depressive disorder. Venlafaxine 631 Study Group. J Clin Psychiatry 61: 95-100.

38. Bielski RJ, Ventura D, Chang CC (2004) A double-blind comparison of escitalopram and venlafaxine extended release in the treatment of major depressive disorder. J Clin Psychiatry 65: 1190-1196.

39. Montgomery SA, Huusom AK, Bothmer J (2004) A randomised study comparing escitalopram with venlafaxine XR in primary care patients with major depressive disorder. Neuropsychobiology 50: 57-64.

40. Lennox-Smith A, Schaeffer P, Reynolds A, Willard L. A double blind trial of venlafaxine XR vs citalopram in patients with treatment resistant depression; 2001; Harrogate, UK.

41. Guelfi JD, Ansseau M, Timmerman L, Korsgaard S (2001) Mirtazapine versus venlafaxine in hospitalized severely depressed patients with melancholic features. J Clin Psychopharmacol 21: 425-431.

42. Gentil V, Kerr-Correa F, Moreno R, D'Arrigo Busnello E, De Campos JA, et al. (2000) Double-blind comparison of venlafaxine and amitriptyline in outpatients with major depression with or without melancholia. J Psychopharmacol 14: 61-66.

43. Perahia DG, Pritchett YL, Kajdasz DK, Bauer M, Jain R, et al. (2008) A randomized, double-blind comparison of duloxetine and venlafaxine in the treatment of patients with major depressive disorder. J Psychiatr Res 42: 22-34.

44. Hacket D, Salinas E, Desmet A. Efficacy and safety of venlafaxine vs fluvoxamine in outpatients with major depression; 1998; Paris, France.

45. Perahia DG, Wang F, Mallinckrodt CH, Walker DJ, Detke MJ (2006) Duloxetine in the treatment of major depressive disorder: a placebo- and paroxetine-controlled trial. Eur Psychiatry 21: 367-378.

46. Goldstein DJ, Lu Y, Detke MJ, Wiltse C, Mallinckrodt C, et al. (2004) Duloxetine in the treatment of depression: a double-blind placebo-controlled comparison with paroxetine. J Clin Psychopharmacol 24: 389-399.

47. Detke MJ, Wiltse CG, Mallinckrodt CH, McNamara RK, Demitrack MA, et al. (2004) Duloxetine in the acute and long-term treatment of major depressive disorder: a placebo- and paroxetine-controlled trial. Eur Neuropsychopharmacol 14: 457-470.

48. Lee P, Shu L, Xu X, Wang CY, Lee MS, et al. (2007) Once-daily duloxetine 60 mg in the treatment of major depressive disorder: multicenter, double-blind, randomized, paroxetine-controlled, non-inferiority trial in China, Korea, Taiwan and Brazil. Psychiatry Clin Neurosci 61: 295-307.

49. Benkert O, Szegedi A, Kohnen R (2000) Mirtazapine compared with paroxetine in major depression. J Clin Psychiatry 61: 656-663.

50. Schatzberg AF, Kremer C, Rodrigues HE, Murphy GM, Jr. (2002) Double-blind, randomized comparison of mirtazapine and paroxetine in elderly depressed patients. Am J Geriatr Psychiatry 10: 541-550.

51. Wade A, Crawford GM, Angus M, Wilson R, Hamilton L (2003) A randomized, double-blind, 24-week study comparing the efficacy and tolerability of mirtazapine and paroxetine in depressed patients in primary care. Int Clin Psychopharmacol 18: 133-141.

52. Boulenger JP, Huusom AK, Florea I, Baekdal T, Sarchiapone M (2006) A comparative study of the efficacy of long-term treatment with escitalopram and paroxetine in severely depressed patients. Curr Med Res Opin 22: 1331-1341.

53. Baldwin DS, Cooper JA, Huusom AK, Hindmarch I (2006) A double-blind, randomized, parallel-group, flexible-dose study to evaluate the tolerability, efficacy and effects of treatment discontinuation with escitalopram and paroxetine in patients with major depressive disorder. Int Clin Psychopharmacol 21: 159-169.

54. Arminen SL, Ikonen U, Pulkkinen P, Leinonen E, Mahlanen A, et al. (1994) A 12-week double-blind multi-centre study of paroxetine and imipramine in hospitalized depressed patients. Acta Psychiatr Scand 89: 382-389.

55. Moon CA, Vince M (1996) Treatment of major depression in general practice: a double-blind comparison of paroxetine and lofepramine. Br J Clin Pract 50: 240-244.

56. Åberg-Wistedt A, Agren H, Ekselius L, Bengtsson F, Akerblad AC (2000) Sertraline versus paroxetine in major depression: clinical outcome after six months of continuous therapy. J Clin Psychopharmacol 20: 645-652.

57. Yoshimura R, Mitoma M, Sugita A, Hori H, Okamoto T, et al. (2007) Effects of paroxetine or milnacipran on serum brain-derived neurotrophic factor in depressed patients. Progress in Neuro-Psychopharmacology and Biological Psychiatry 31: 1034-1037.

58. Laurelle M, Reynaert C, Collin A, Goffinet S, Seghers A, et al. (1991) A multicenter double-blind comparative study between paroxetine and maprotiline in major depression. Eur Neuropsychopharmacol 1: 439S.

59. Nierenberg AA, Greist JH, Mallinckrodt CH, Prakash A, Sambunaris A, et al. (2007) Duloxetine versus escitalopram and placebo in the treatment of patients with major depressive disorder: onset of antidepressant action, a non-inferiority study. Curr Med Res Opin 23: 401-416.

60. Jonas J, Bose A, Alexopoulos G, Gommoll C, Li D, et al. (2006) Double-blind comparison of escitalopram and duloxetine in the acute treatment of major depressive disorder. Neuropsychopharmacology 31 (supl): S166.

61. Wade A, Gembert K, Florea I (2007) A comparative study of the efficacy of acute and continuation treatment with escitalopram versus duloxetine in patients with major depressive disorder. Curr Med Res Opin 23: 1605-1614.

62. Khan A, Bose A, Alexopoulos GS, Gommoll C, Li D, et al. (2007) Double-blind comparison of escitalopram and duloxetine in the acute treatment of major depressive disorder. Clin Drug Investig 27: 481-492.

63. Colonna L, Andersen HF, Reines EH (2005) A randomized, double-blind, 24-week study of escitalopram (10 mg/day) versus citalopram (20 mg/day) in primary care patients with major depressive disorder. Curr Med Res Opin 21: 1659-1668.

64. Moore N, Verdoux H, Fantino B (2005) Prospective, multicentre, randomized, double-blind study of the efficacy of escitalopram versus citalopram in outpatient treatment of major depressive disorder. Int Clin Psychopharmacol 20: 131-137.

65. Lepola UM, Loft H, Reines EH (2003) Escitalopram (10-20 mg/day) is effective and well tolerated in a placebo-controlled study in depression in primary care. Int Clin Psychopharmacol 18: 211-217.

66. Ventura D, Armstrong EP, Skrepnek GH, Haim Erder M (2007) Escitalopram versus sertraline in the treatment of major depressive disorder: a randomized clinical trial. Curr Med Res Opin 23: 245-250.

67. Van Amerongen AP, Ferrey G, Tournoux A (2002) A randomised, double-blind comparison of milnacipran and imipramine in the treatment of depression. J Affect Disord 72: 21-31.

68. Tignol J, Pujol-Domenech J, Chartres JP, Leger JM, Pletan Y, et al. (1998) Double-blind study of the efficacy and safety of milnacipran and imipramine in elderly patients with major depressive episode. Acta Psychiatr Scand 97: 157-165.

69. Behnke K, Sogaard J, Martin S, Bauml J, Ravindran AV, et al. (2003) Mirtazapine orally disintegrating tablet versus sertraline: a prospective onset of action study. J Clin Psychopharmacol 23: 358-364.

70. Rossini D, Serretti A, Franchini L, Mandelli L, Smeraldi E, et al. (2005) Sertraline versus fluvoxamine in the treatment of elderly patients with major depression: a double-blind, randomized trial. J Clin Psychopharmacol 25: 471-475.

71. Wisner KL, Hanusa BH, Perel JM, Peindl KS, Piontek CM, et al. (2006) Postpartum depression: a randomized trial of sertraline versus nortriptyline. J Clin Psychopharmacol 26: 353-360.

72. Keller MB, Gelenberg AJ, Hirschfeld RM, Rush AJ, Thase ME, et al. (1998) The treatment of chronic depression, part 2: a double-blind, randomized trial of sertraline and imipramine. J Clin Psychiatry 59: 598-607.

73. Haffmans PM, Timmerman L, Hoogduin CA (1996) Efficacy and tolerability of citalopram in comparison with fluvoxamine in depressed outpatients: a double-blind, multicentre study. The LUCIFER Group. Int Clin Psychopharmacol 11: 157-164.

74. Kyle CJ, Petersen HE, Overo KF (1998) Comparison of the tolerability and efficacy of citalopram and amitriptyline in elderly depressed patients treated in general practice. Depress Anxiety 8: 147-153.

75. Birkenhager TK, van den Broek WW, Mulder PG, Bruijn JA, Moleman P (2004) Comparison of two-phase treatment with imipramine or fluvoxamine, both followed by lithium addition, in inpatients with major depressive disorder. Am J Psychiatry 161: 2060-2065.

76. Schwartz G, Such P, Schatzberg A (2002) Reboxetine vs venlafaxine in the treatment of severe major depression European Neuropsychopharmacology 12 Suppl 3: S204.

77. Blier P, Gobbi G, Turcotte JE, de Montigny C, Boucher N, et al. (2009) Mirtazapine and paroxetine in major depression: a comparison of monotherapy versus their combination from treatment initiation. Eur Neuropsychopharmacol 19: 457-465.
