# Supplementary material for: Effectiveness and Cost-Effectiveness of Antidepressants in Primary Care: A Multiple Treatment Comparison Meta-Analysis and Cost-Effectiveness Model
Source: PLoS One. 2012 Aug 2;7(8):e42003. doi: 10.1371/journal.pone.0042003 (PMC3410906; doi:10.1371/journal.pone.0042003)
Supplement: Table S3 — Frequency of drop outs due to adverse events. (DOCX) [file pone.0042003.s003.docx]

| **Drug** | | **Frequency drop out** | **Confidence interval** | |
| --- | --- | --- | --- | --- |
| Flouxetin | | 0.0778 | 0.0633 | 0.0940 |
| Venlafaxin | | 0.1049 | 0.0807 | 0.1337 |
| Paroxetin | | 0.1046 | 0.0743 | 0.1404 |
| Mirtazapin | | 0.0779 | 0.0526 | 0.1104 |
| Escitalopram | | 0.0631 | 0.0426 | 0.0880 |
| Duloxetin | | 0.1493 | 0.1086 | 0.1985 |
| Imipramin | | 0.1063 | 0.0600 | 0.1705 |
| Citalopram | | 0.0904 | 0.0515 | 0.1455 |
| Sertralin |  | 0.0550 | 0.0298 | 0.0898 |
| Amitriptylin | | 0.1446 | 0.0813 | 0.2333 |
| Fluvoxamin | | 0.1573 | 0.0676 | 0.3024 |
| Klomipramin | | 0.4013 | 0.1586 | 0.7126 |
| Dothiepin |  | 0.0517 | 0.0089 | 0.1486 |
| Lofepramine | | 0.0955 | 0.0158 | 0.2715 |
| Milnicipran | | 0.0467 | 0.0133 | 0.1101 |
| Maprotilin |  | 0.0007 | 0.0000 | 0.0063 |
| Reboxetin | | 0.1063 | 0.0312 | 0.2396 |
